# Nephrology intervention to avoid acute kidney injury in patients awaiting cardiac surgery: randomized clinical trial

**DOI:** 10.3389/fneph.2024.1470926

**Published:** 2024-11-13

**Authors:** Sergi Codina, Laia Oliveras, Eva Ferreiro, Aroa Rovira, Ana Coloma, Nuria Lloberas, Edoardo Melilli, Miguel Hueso, Fabrizio Sbraga, Enric Boza, José M. Vazquez, José L. Pérez-Fernández, Joan Sabater, Josep M. Cruzado, Nuria Montero

**Affiliations:** ^1^ Nephrology, Hospital de Bellvitge, Barcelona, Spain; ^2^ Nephrology, Idibell, Barcelona, Spain; ^3^ Nephrology, Hospital de Vinaros, Vinaros, Spain; ^4^ Cardiac Surgery, Hospital de Bellvitge, Barcelona, Spain; ^5^ Anesthesiology, Hospital de Bellvitge, Barcelona, Spain; ^6^ Anesthesiology Department, Hospital Universitari de Vall Hebrón, Barcelona, Spain; ^7^ Intensive Care Unit, Hospital de Bellvitge, Barcelona, Spain

**Keywords:** acute kidney injury, cardiac surgery, nephrology intervention, clinical trials, intensive care

## Abstract

**Introduction:**

Cardiac surgery-associated acute kidney injury (CSA-AKI) is a well-known complication that increases morbidity and mortality rates. The objective of this study was to reduce CSA-AKI through nephrologist intervention in patients awaiting cardiac surgery.

**Methods:**

We performed a single center, open-label, randomized clinical trial including 380 patients who underwent scheduled cardiac surgery at the Hospital de Bellvitge between July 2015 and October 2019. A total of 184 patients were evaluated by the same Nephrologist one month before the surgery to minimize the risk factors for AKI. In addition to assessments at the outpatient clinic, we also collected clinical data during hospitalization and during the first year.

**Results:**

Despite the intervention, no differences were observed between the groups in the incidence of CSA-AKI (intervention group 26.37% vs. standard of care 25.13%, p=0.874), mortality (3.91% vs. 3.59%, p=0.999), length of Intensive Care Unit (ICU) stay (10 days [7.00;15.0] for both groups, p=0.347), or renal function after one year of follow-up (estimated glomerular filtration rate (eGFR) by CKD-EPI: 74.5 ml/min (standard deviation 20.6) vs 76.7 (20.8) ml/min, respectively, p=0.364). A reduction in the need for blood transfusion was observed in the intervention group, although the difference was not statistically significant (37.22% vs. 45.03%, p =0.155).

**Conclusion:**

In this clinical trial, nephrologist intervention in the entire population on the cardiac surgery waiting list did not show a nephroprotective benefit.

**Clinical trial registration:**

ClinicalTrials.gov, identifier (NCT02643745).

## Introduction

Acute kidney injury (AKI) is a common complication of cardiac surgery. In the literature, the incidence varies from 7 to 44% depending on the study. There are different definitions used: AKIN classification (Acute Kidney Injury Network), RIFLE criteria (Risk, Injury, Failure, Loss, End-Stage Kidney Disease), or KDIGO (Kidney Disease Improving Global Outcomes) criteria based on serum creatinine and urine output (1–4). The presence of AKI has a clinical impact because it is associated with longer hospitalization, progression to end-stage renal disease (ESRD), and even increased mortality (2). The mortality risk in patients who develop acute renal dysfunction after cardiac surgery increases by approximately 40%, ranging from 2% to 19% according to the series (5).

There are some well-known risk factors associated with AKI, including baseline patient characteristics (age and comorbidities), need for perioperative blood transfusion, and the presence of earlier chronic kidney disease (6, 7). For many years, different interventions designed to prevent post-surgical AKI have been attempted without success (8). In contrast, a holistic approach during the post-operative period, guided by the implementation of the Kidney Disease Improving Global Outcomes (KDIGO) bundle of care in patients at high risk of AKI, has shown a reduction in AKI incidence, especially a decrease in moderate and severe AKI (9).

However, the prevention of AKI related to surgery should begin prior to the operation since most risk factors are modifiable. According to the ADQI (Acute Disease Quality Initiative), all patients undergoing cardiac surgery should undergo routine clinical assessment of AKI risk to implement preventive strategies (10).

The aim of this randomized clinical trial was to assess whether nephrology intervention before cardiac surgery can reduce the postoperative incidence of AKI.

## Methods

### Trial design and participants

We conducted a single-center, open-label, randomized clinical trial that included patients on the waiting list for cardiac surgery. Eligible patients were adults aged > 18 years who were awaiting scheduled cardiac surgery. Exclusion criteria were requirement for renal replacement therapy (RRT) before surgery, need for urgent surgery, or participation in another clinical trial. Informed consent to participate in the study was obtained from all participants.

The Clinical Research Ethics Committee of the Hospital de Bellvitge approved this study before its initiation. We followed the CONSORT guidelines to report this RCT study, and the protocol was published at clinicaltrials.gov (NCT02643745).

### Randomization and intervention

Patients were randomly assigned to the nephrology-intervention or control group (1:1) using a computer random number generator. Eligible patients were distributed using sequentially numbered opaque sealed envelopes during the first visit of the cardiac surgeon.

The patients assigned to the nephrologist intervention group (intervention group) had a preoperative and protocolized study that included blood and urine tests, bioimpedance at consultation and before surgery, and an outpatient clinic visit with a nephrologist who performed a detailed anamnesis, detected risk factors, and improved the overall patient’s condition (**Supplementary Table 2**). Patients assigned to the no-intervention group (standard of care) followed the usual routine care and did not receive any other consultation by a nephrologist, cardiologist or cardiac surgeon before surgery. Bioimpedance was performed in a subgroup of patients before surgery.

After hospital discharge, all patients visited the Nephrology Department. Clinical and analytical assessments were performed at discharge and 6 and 12 months after surgery. All consultations were conducted by the same nephrologist.

### Outcomes

The primary endpoint was the presence of cardiac surgery-associated AKI (CSA-AKI). We defined according to the KDIGO criteria (**Supplementary Table 1**) (11). The secondary endpoints were mortality (in the first year), hospitalization days, length of stay in (Intensive Care Unit (ICU), need for RRT, anemia, need for blood transfusion, metabolic disorder control (diabetes, dyslipidemia), and renal function using the estimated glomerular filtration rate (eGFR) calculated by the Chronic Kidney Disease Epidemiology Collaboration (CKD-EPI).

We collected clinical data at randomization, before and after surgery, during hospitalization, and 6 and 12 months after surgery. Data were collected from the medical records. It included: baseline characteristics (age, ethnicity, gender, body mass index (BMI), comorbidities, and treatments); blood and urine analysis (serum creatinine (SCr), albuminuria (using urine albumin-to-creatinine ratio (ACR) in a spot urine sample), and proteinuria (using protein-to-creatinine ratio (PCR) in a spot urine sample), urinary ionogram, ferritin, transferrin saturation index, albumin, prealbumin, cholesterol levels (low-density lipoprotein (LDL), high-density lipoprotein (HDL), and total), fibrinogen, fasting blood glucose, HbA1c and hemoglobin, venous blood gasometry); spectroscopic bioimpedance analysis measuring: lean tissue index (LTI), fat tissue index (FTI), normohydrated weight, and the distribution of fluids in the body (total body water (TBW), extracellular water (ECW) and intracellular water (ICW) using BCM-Body Composition Monitor (Fresenius)); surgery characteristics (type of surgery, individual severity risk, off-pump time surgery, clamp time, need of post-operative pacemaker, intraoperative hemodynamics, drug use, fluid balance, and need of transfusions); and post-operative evolution (need of post-operative pacemaker, hemodynamics, drug use, fluid balance, the need of transfusion and type and duration of RRT if needed).

### Statistical analysis

We aimed to detect a clinical difference of 5% reduction in the incidence of AKI between the study groups, with an expected rate of 12.2% AKI in the control group. A total of 550 subjects per group were required to have 80% power to reject the null hypothesis that the AKI rate was the same in the experimental and control groups. The type I error probability associated with this test was 5%. The investigators planned interim analyses after 25, 50%, and 75% of the trial participants completed their 1-year follow-up. However, after the 410 patients were randomized, it became apparent that the rate of the primary outcome was higher than expected in both groups. This made the trial underpowered to detect a 5% reduction in AKI rate. In fact, the trial would only be powered to detect differences of > 10%. An effect that was considered unattainable with an intervention such as that planned in our trial. Therefore, the trial monitoring committee decided to stop the trial in October 2019.

We performed an intention-to-treat analysis considering the results of the patient in the first assigned group. Continuous variables were compared between groups using the Student’s t-test or Wilcoxon rank test according to distribution. Categorical variables were compared using the X^2^ or Fisher’s exact test, when appropriate. We performed subgroup analysis for patients at risk based on the presence of eGFR ≤ 45 ml/min/1.73 m^2^. Univariate and multivariate logistic regression models were used to estimate factors associated with AKI. Results are reported as odds ratios (OR) and 95% confidence intervals (CIs). All analyses were performed with a two-sided significance level of 0.05 and were conducted with SPSS software and R software version 4.1.0 [The Comprehensive R Archive Network. Available from: https://cran.r-project.org/].

## Results

**Figure 1:**
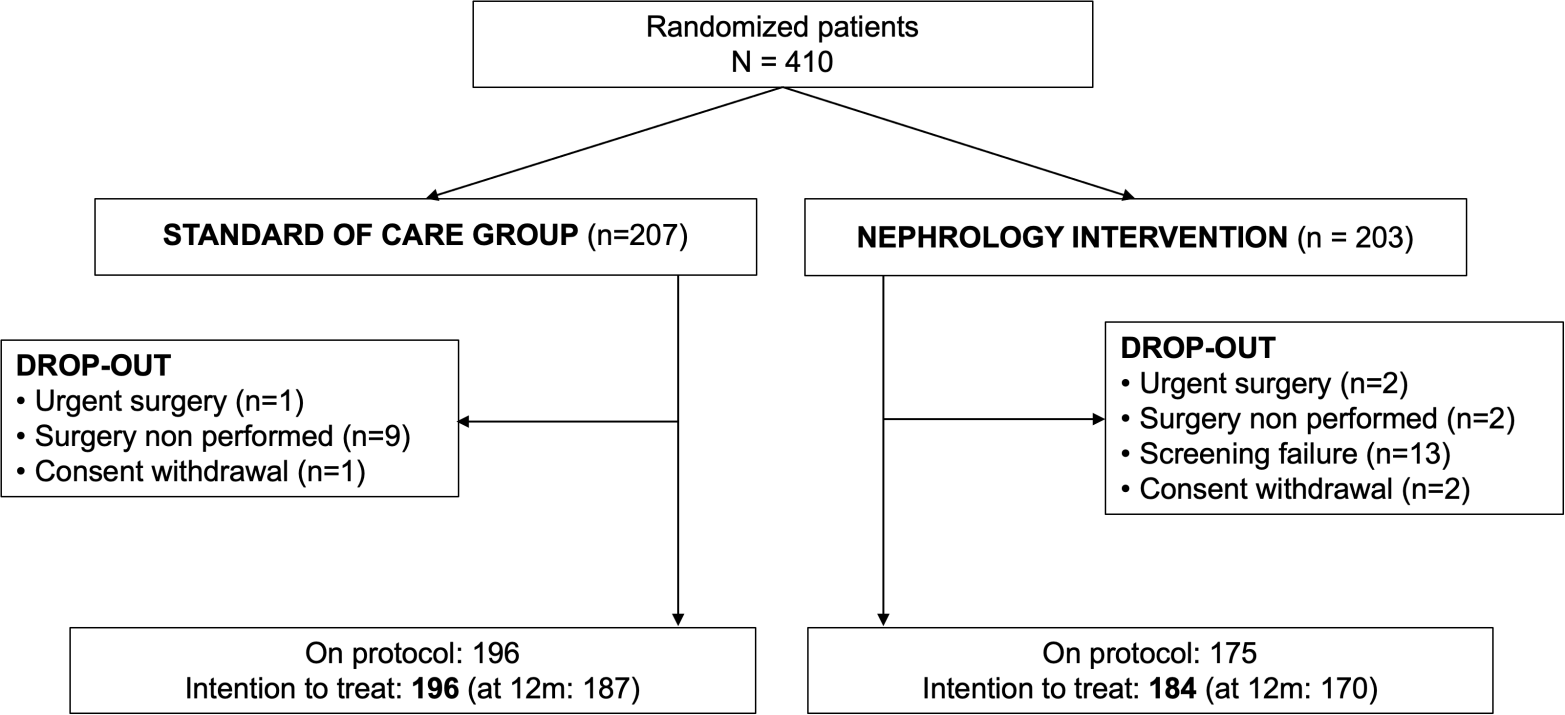
Flowchart of included patients.

From July 2015 to October 2019, 410 patients who underwent cardiac surgery at the Hospital de Bellvitge were included in the clinical trial. A total of 203 participants were assigned to the intervention group and 207 to the standard care group. After excluding 30 patients, 380 were finally analyzed: 184 in the intervention group and 196 in the standard care group (n=196, **Figure 1**). The baseline characteristics of both groups did not differ significantly, except for more smokers (7.27% vs. 1.68%), chronic obstructive pulmonary disease (21.9% vs. 13%), and vascular disease (13.1% vs. 7.7%) in the intervention group (**Table 1**). Most surgeries were of one isolated valve (65.3%). There were no significant differences between the groups in terms of surgery type or perioperative management of volume or drugs (**Table 1**). At the time of surgery, no differences were observed in spectroscopic bioimpedance analysis parameters (**Supplementary Table 2**).

**Table 1:** Characteristics of the participants and surgery, according to Group.

	Nephrology intervention n= 184	Standard of care n =196
Participant characteristics at Baseline		
Age (years, mean (sd))	66.2 (13.8)	66.2 (11.4)
Female Sex (n, %)	60 (32.8)	74 (37.8)
Weight (Kg, mean (sd))	76.3 (13.9)	76.6 (13.7)
BMI (Kg/m^2^, mean (sd))	28.1 (4)	28 (3.97)
Smoker yes; Previous (n, %)	12 (7.3); 33 (20)	3 (1.7); 8 (4.5)
Use of alcohol, n (%)	18 (9.8)	12 (6.2)
Charlson index (mean (sd))	2.01 (1.9)	1.75 (1.8)
Type 1 diabetes (n, %)	1 (0.6)	3 (1.5)
Type 2 diabetes (n, %)	48 (26.2)	49 (25.3)
Hypertension (n, %)	137 (73.6)	131 (66.8)
Stroke (n, %)	13 (7.1)	14 (7.3)
Cardiac disease (n, %)	65 (35.9)	59 (30.6)
COPD (n, %)	40 (21.9)	25 (13)
CKD, (n, %)	32 (17.5)	27 (14)
Solid organ cancer, (n, %)	18 (9.9)	16 (8.2)
Hematological cancer (n, %)	4 (2.2)	3 (1.6)
Liver disease (n, %)	14 (7.7)	9 (4.7)
Vasculopathy (n, %)	24 (13.1)	15 (7.8)
Charlson index (mean, sd)	2.01 (1.9)	1.75 (1.8)
Surgery characteristics		
Type of surgery (n, %) Bentall-de Bono Aortocoronary bypass Interatrial communication David Procedure Aortic reparation Aortic prothesis David + Aortic prothesis Mitral prothesis Mitral reparation Tricuspid reparation Double valve intervention	4 (2.2) 36 (20) 1 (0.6) 5 (2.8) 4 (2.2) 66 (36.7) 5 (2.8) 35 (19.4) 18 (10) 2 (1.1) 4 (2.2)	8 (4.2) 38 (20.1) 2 (1.1) 5 (2.7) 9 (4.7) 67 (35.4) 12 (6.4) 26 (13.8) 19 (10) 2 (1.1) 1 (0.5)
Surgery by complexity (n, %) * Simple One isolated valve Two valves Complex	37 (20.6) 125 (69.4) 4 (2.2) 14 (7.8)	40 (21.2) 123 (65.1) 1 (0.5) 25 (13.2)
Antibiotic (n, %) Cefuroxime Teicoplanin Tobramycin Vancomycin None	167 (93.9) 3 (1.7) 1 (0.6) 6 (3.4) 1 (0.6)	174 (92.1) 2 (1.1) 2 (1.1) 11 (5.8) 0
Clamp time (minutes, mean (sd))	69.2 (39.4)	72 (43.1)
Transfusion (units, mean (sd) Blood Platelets Plasma	0.25 (0.7) 0.18 (0.5) 0.06 (0.5)	0.29 (0.7) 0.24 (0.6) 0.11 (0.5)
SAP begin (mmHg, mean (sd))	111 (16.4)	113 (15.7)
SAP end (mmHg, mean (sd))	108 (13.4)	108 (14.2)
DAP begin (mmHg, mean (sd))	57.4 (10.7)	57.9 (9.9)
DAP end (mmHg, mean (sd))	58.2 (10.1)	57.5 (9.9)
HR begin (beats per minute, mean(sd))	73.4 (15.1)	72.8 (14.7)
HR end (beats per minute, mean (sd))	82.1 (12.7)	82.6 (15)
CVP begin (mmHg, mean (sd))	7.8 (3.02)	9.3 (3.2)
CVP end (mmHg, mean (sd))	8.8 (2.8)	9.5 (3.3)

BMI, Body Mass Index; Cardiac disease, History of structural or ischemic heart disease other than surgical disease; CKD, Chronic kidney disease; COPD, Chronic obstructive pulmonary disease; CVP, Central venous pressure; DAP, diastolic blood pressure; HR, heart rate; Kg, kilograms; SAP, systolic blood pressure; SD,Standard deviation.

*Simple: coronary bypass, closure of interatrial communication. One isolated valve: aortic prosthesis, aortic repair, mitral prosthesis, mitral repair, tricuspid repair. Two valves. Complex: combined replacement of aortic valve and ascending aorta, Bentall-de Bono surgery, David procedure.

### Nephrology intervention

In the 184 patients randomized into the intervention group, the nephrology consultant indicated interventions in 15 distinct categories related to renal health following the KDIGO guidelines for AKI (**Table 2**; **Supplementary Table 3**). This Nephrology Intervention was performed a minimum of one month before surgery (median 54 days; interquartile range 31.75-82). The most common interventions were the reduction of caloric intake (n=71), diuretic and antihypertensive treatment adjustment (n=29, n=41), and iron correction (n=32). Intervention achievement was evaluated at patient admission before surgery and 6 and 12 months after surgery. The median number of interventions indicated was 2 per patient (interquartile range IQR 1-3) with a mean global achievement per patient of 70.3 ± 36.1%. Despite a good accomplishment rate, no differences were observed in those variables that could be measured at the time of randomization with respect to the time of admission to the hospital between the standard-of-care and Nephrology Intervention Groups (**Supplementary Table 4**).

**Table 2:** Nephrology interventions and accomplishment.

	Number of proposed interventions (n of patients, %)	Total accomplishment (%)
Acidosis correction	1 (0.6)	1 (100)
Diet assessment	71 (40.3)	57 (40.3)
Tobacco assessment	15 (8.5)	6 (40)
Glycaemia assessment	17 (9.7)	10 (58.8)
Obesity reduction	76 (43.2)	25 (32.9)
Proteinuria reduction	3 (1.7)	3 (100)
Salt intake assessment	12 (6.8)	10 (8.3)
Diuretic adjustment Reduction/Withdraw Initiation/Increase	17 (9.7)/8 (4.6) 3 (1.7)/1 (0.6)	28 (96.6)
Statin adjustment	33 (19.1)	30 (93.8)
ACEi or ARBs adjustment Reduction/Withdrawal Initiation/Increase	7 (3.9)/1 (0.6) 7 (3.9)/5 (2.8)	20 (100)
Other antihypertension drugs adjustment Drug initiation Drug withdraw	22 (12.6) 19 (10.6)	38 (92.7)
Antiplatelet adjustment	5 (2.8)	5 (100)
Hypouricemia drugs initiation	8 (4.6)	6 (75)
NSAID withdraw	16 (9.1)	15 (93.8)
Anemia assessment Iron therapy Blood transfusion	32 (18.2) 1 (0.6)	28 (84.8)

ACEi, Angiotensin-converting enzyme inhibitors; ARBs, Angiotensin II receptor blockers; NSAID, Non-steroidal anti-inflammatory drugs.

### Acute kidney injury

The overall incidence of CSA-AKI was 25.73%, without differences between the groups (26.37% in intervention vs. 25.13% in standard-of-care, p=0.874). Most episodes of CSA-AKI were staged as AKI 1 (18.6% vs. 17.4%). In contrast, stage 3 AKI was marginal in both groups (3.83% and 1.54%, respectively). Although AKI stage 2 was less frequent in the intervention group, the difference was irrelevant (3.83% vs. 6.15%).

In the univariate analysis, the risk factors associated with CSA-AKI were age (odds ratio (OR) 1.05; 95% confidence interval (CI) 1.03 to 1.08; p<0.001), BMI (OR 1.06; 95% CI 1.00 to 1.13; p=0.034), clamp time (OR 1.01, 95% CI 1.00 to 1.01; p=0.026), and previous chronic kidney disease (OR 3.50; 95% CI 1.96 to 6.26; p<0.001). The factors associated with a lower risk of CSA-AKI were diastolic pressure at the beginning of surgery (OR 0.97; 95% CI 0.95 to 0.99; p=0.009), diastolic pressure at the end of the clamp (OR 0.96; 95% CI 0.94 to 0.99; p=0.004), and hemoglobin levels at hospital admission (OR 0.98; 95% CI 0.97 to 1.00; p=0.019, **Table 3**). In multivariate analysis, age, BMI, previous chronic kidney disease, and clamp time remained significant (**Table 4**).

**Table 3:** Association of main clinical, analytical and surgery characteristics with acute kidney injury.

	Acute kidney injury		
	No (n=280)	Yes (n=97)	p-value
Clinical characteristics			
Age (years, mean (sd))	64.7 (13.3)	70.5 (9.1)	<0.001
Sex (n, %): Male/Female	181/99 (64.6/35.4)	64/33 (66/34)	0.82
Body mass index (Kg/m^2^, mean (sd))	27.8 (3.9)	28.8 (4.2)	0.04
Comorbidities (n, %):			
• Stroke	21 (7.6)	6 (6.2)	0.66
• Type 1 diabetes	3 (1.1)	1 (1.1)	0.96
• Type 2 diabetes	72 (25.8)	24 (25)	0.89
• Cardiac disease	95 (34.4)	29 (30.2)	0.46
• Pulmonary disease	46 (16.6)	19 (19.6)	0.50
• Active cancer	23 (8.2)	11 (11.5)	0.35
• Use of alcohol	23 (8.2)	7 (7.2)	0.78
• Hypertension	191 (68.5)	72 (74.2)	0.29
• Chronic Kidney Disease	30 (10.8)	29 (29.9)	<0.001
• Solid organ cancer	23 (8.2)	11 (11.5)	0.35
• Hematological cancer	5 (1.8)	2 (2.1)	0.83
• Liver disease	19 (6.9)	4 (4.1)	0.35
• Vasculopathy	29 (10.5)	10 (10.3)	0.98
Surgery characteristics			
High risk surgery* (n, %)	31 (11.1)	13 (13.4)	0.67
Clamp time (minutes, mean (sd))	67.4 (39.2)	78.4 (43.1)	0.03
SAP (mmHg mean (sd)) begin/end	112(15.5)/ 108 (14)	112 (17.6)/ 108 (13)	0.94/0.98
DAP (mmHg mean (sd)) begin/end	58.6 (9.5)/58.9 (9.7)	55.3 (11.9)/55.3 (9.78)	0.01/0.004
Pre-surgery laboratory parameters			
Serum creatinine (μmol/mL, mean (sd))	82.4 (24.9)	91.6 (35.9)	0.01
GFR categorized ≥45 mL/min/1.73m^2^ (n, %)	11 (4.6)	14 (15.6)	0.002
Haemoglobin (g/dL, mean (sd))	133 (16.1)	128 (20.1)	0.02
Hospitalization potential kidney injuries (n (%))			
Vancomycin use	12 (4.5)	5 (5.2)	–
Iodinated contrast media use	13 (5.2)	5 (6.2)	0.73
ACEi use	140 (50)	46 (47.4)	0.87

*High risk surgeries were: Double valve intervention, combined replacement of aortic valve and ascending aorta, Bentall-de Bono surgery, David procedure surgery.

ACEi, Angiotensin-converting enzyme inhibitors; DAP, dyastolic blood pressure; GFR, Glomerular filtration rate by CKD-EPI formulae; SAP, systolic blood pressure.

**Table 4:** Risk factors associated with acute kidney injury.

	Univariate OR (95% CI)	p-value	Multivariate OR (95% CI)	p-value
Age	1.05 [1.03;1.08]	<0.001	1.05 (1.02; 1.08)	0.001
Body mass index	1.06 [1.00;1.13]	0.034	1.07 (1.00; 1.14)	0.043
Chronic kidney disease	3.50 [1.96;6.26]	<0.001	3.04 (1.62; 5.72)	0.001
Clamp time	1.01 [1.00;1.01]	0.026	1.01 (1.00; 1.01)	0.037
DAP begin	0.97 [0.95;0.99]	0.009	–	–
DAP end	0.96 [0.94;0.99]	0.004	–	–

DAP, diastolic blood pressure; CI, Confidence Interval; OR, Odds Ratio.

We then evaluated whether the nephrologist intervention had any impact on a selected group of patients at risk of CSA-AKI based on the presence of eGFR ≤ 45 ml/min/1.73 m^2^. In the high-risk patients randomized to the intervention group (n=13), we found a lower incidence of AKI (46.15% vs. 66.67%), although the differences did not reach statistical significance (p=0.53), probably because of the low number of high-risk patients (n=25). When the selection of patients at risk of CSA-AKI was analyzed according to the type of surgery (high versus low risk), no differences were observed in either group (high risk surgery 27.78% vs 30.77%, low risk surgery 26.22% vs 24.26%).

### Secondary outcomes

Regarding secondary outcomes, there were no differences in terms of mortality between the groups, neither during admission (2.17% vs. 2.04%, p =0.999) nor in the first year after surgery (3.91% vs. 3.59%, p=0.999). A reduction in the need for blood transfusion was observed in the intervention group, although the difference was not statistically significant (37.22% vs. 45.03%, p =0.155). Only four patients required RRT (intervention group, n=3; control group, n=1). The median length of hospitalization was 10 days [range, 7–15] days) in both the groups (p=0.347).

When long-term outcomes were evaluated, we did not find any differences in eGFR between the intervention group and the standard care group (74.5 ± 20.6 ml/min vs 76.7 ± 20.8 ml/min respectively, p=0.364).

No differences were observed in blood cholesterol levels at 6 months after surgery (4.32 ± 0.98 vs 4.44 ± 1.09 mmol/L; p=0.524) and after 12 months (4.53 ± 1.01 vs 4.49 ± 1.16 mmol/L; p=1), neither in glycosylated hemoglobin levels (at 6 months: 5.69 ± 0.75 vs 5.7 ± 0.72%, p=0.903 and at 12 months: 6.19 ± 1.17 vs 6.24 ± 1.08%, p=0.748).

## Discussion

In this randomized controlled trial, a nephrology intervention to avoid acute kidney injury after cardiac surgery did not show any benefit at the clinical level.

These results are surprisingly contrary to earlier findings. Recent studies have shown that the application of the KDIGO bundle of care protocols in early ICU hospitalization prevents moderate-to-severe AKI (9). Nevertheless, according to the ADQI (Acute Disease Quality Initiative), as many of the risk factors are modifiable, all patients undergoing cardiac surgery should undergo routine clinical assessment of AKI risk to implement preventive strategies before the operation (10). However, in our study, a nephrologist intervention focused on the correction of these potential AKI risk factors one month before surgery was not associated with the prevention of AKI and did not support these recommendations.

It is worth noting that the incidence of CSA-AKI in our cohort was lower than that previously described in some series in the literature. For example, in the PrevAKI study (9) the incidence of AKI was 55% and 71%, while in our cohort, the incidence was approximately 25% for both groups. In a study by Silva et al (12), the incidence of AKI was 43% and was associated with increased mortality, regardless of the grade of AKI. Howitt et al (2) study, the incidence of AKI can reach up to 36.1%. Many other recently published case series reported incidences of CSA-AKI similar to those observed in our study (13–16). Even in the TRIBE-AKI study (17), the incidence was only 30%, although the design of the study selected high-risk patients with CSA-AKI. It is important to note that these differences may be due to the different diagnostic criteria for AKI, which can vary significantly depending on the definition used because some only assess serum creatinine and others also take into account urine output criteria (1).

From our point of view, the differences observed in terms of CSA-AKI are related to the quality of post-operative care, especially in the first few hours in the ICU. In fact, the “intervention” that demonstrates benefits in the PrevAKI study (9) is nothing more than applying good clinical practices for the care of renal function. All these practices (consisting of avoidance of nephrotoxic agents, discontinuation of angiotensin-converting enzyme inhibitors and angiotensin receptor blockers for the first 48 h after surgery, close monitoring of serum creatinine and urine output, avoidance of hyperglycemia for the first 72 h after surgery, consideration of alternatives to radiocontrast agents, and close hemodynamic monitoring using a prespecified algorithm) should be part of routine clinical practice in all ICUs that treat patients with this high level of complexity.

AKI staging has a significant impact on the subsequent outcomes (2, 18). In our case, for example, most AKI episodes were classified as AKI stage I (70%); therefore, we did not observe a significant deterioration in renal function one year after the surgery in these patients, consistent with that previously reported (18, 19). Based on our results, we must assume that the current state of CSA-AKI is as follows: the incidence of AKI-CSA is approximately 25% in most cases associated with mild stages of AKI (stage II) that do not represent significant differences in mortality or subsequent CKD development.

The risk factors for CSA-AKI seen in our population have also been described in previous reports: the presence of earlier chronic kidney disease, older age, higher clamp time, and lower hemoglobin levels at hospitalization.

Contrary to what is recommended by the Acute Disease Quality Initiative (10), in our case, a nephrological intervention implemented in almost 200 patients did not result in a significant change in subsequent renal outcomes (CSA-AKI in the intervention group: 26.37% vs. 25.13% in the control group). On the other hand, it has meant an increase in work at the nephrologist’s office, which leads to an increase in hospital costs.

The aim of the intervention was to control the main AKI risk factors previously described (6, 7), and the median achievement of the interventions was 70.3%, which we considered positive. However, our intervention did not have a clinically significant impact. For example, at the nephrologist visit, an assessment was made of the degree of anemia and iron deficiency of the patients, given the relationship between iron metabolism and the risk of CSA-AKI (20, 21). This intervention achieved a decrease in the need for transfusion of red blood cell concentrates, but this difference did not reach statistical significance, and it did not impact the main outcome either. Therefore, we do not believe that the absence of differences observed in the study is due to the design or execution of the nephrological intervention, but to the low current impact of CSA-AKI on clinical outcomes in the general population compared to what was expected.

Currently, there is a trend towards the development of predictive tools for CSA-AKI either through “machine learning” tools (14, 22), scores based on clinical data (13) or the discovery of pre- or post-operative biomarkers (15, 17, 20, 23, 24). The general objective of these lines of research is to identify, as accurately as possible, subgroups of the population at a higher risk of AKI and post-surgical complications. For example, in a study by our group, we described how previous chronic kidney disease conferred a pro-inflammatory state that meant an increased susceptibility to the risk of developing CSA-AKI (25).

### Limitations

Arguably, the presence of higher comorbidity in the nephrology intervention group could have masked the study results. However, we believe that the impact of the observed differences in comorbidities (smokers, COPD, vascular disease) should be small as they have little impact on the Charlson index. In addition, it is possible that a thorough anamnesis by an expert nephrologist may have helped to unmask some diagnoses and slightly explain some of the differences found.

The main limitation of our study was the inability to reach the initial sample size, mainly due to the low incidence of CSA-AKI. However, our study features a number of strengths, in particular, our study is the first randomized clinical trial that applies a systematized pre-surgical nephrology intervention with the aim of reducing CSA-AKI in a final cohort of more than 400 patients. The current work provides novel insights by providing a clear message: with a good follow-up of the clinical guidelines, it is not necessary for all patients to be visited by a nephrologist.

Although we advise against the routine evaluation of all patients before cardiac surgery, in the future, it will be possible to consider an intervention only for a selected high-risk subgroup of patients, either in the pre- or post-surgical period. Indeed, we observed a trend toward minimization of CSA-AKI when we evaluated high-risk patients, even though the study was not designed for this purpose and there was not enough sample size to show a significant result.

## Conclusions

Performing a Nephrological Intervention on all patients on the cardiac surgery waiting list has not shown a benefit in reducing CSA-AKI, partly because of the low impact of this entity on these patients when good clinical practices are applied. The identification of groups at increased risk of CSA-AKI may provide an opportunity to carry out personalized interventions in this specific group of patients.

## Data Availability

The original contributions presented in the study are included in the article. Further inquiries can be directed to the corresponding authors.
