# Supplementary material for: Nephrology intervention to avoid acute kidney injury in patients awaiting cardiac surgery: randomized clinical trial
Source: Front Nephrol. 2024 Nov 13;4:1470926. doi: 10.3389/fneph.2024.1470926 (PMC11599165; doi:10.3389/fneph.2024.1470926)
Supplement: Supplementary file 1 [file Table1.docx]

**SUPPLEMENTAL MATERIAL**

**Supplemental Table 1: Kidney Disease Improving Global Outcomes (KDIGO) definition of acute kidney injury.**

| Stage | Serum creatinine criteria | Urine output criteria |
| --- | --- | --- |
| 1 | Rise in serum creatinine ≥26.5 μmol/L in 48 h, or rise 1.5-1.9 times from baseline | 0.5 mL/kg/h for 6-12 h |
| 2 | Rise in serum creatinine 2.0-2.9 times from baseline | 0.5 mL/kg/h for ≥12 h |
| 3 | Rise in serum creatinine 3 times from baseline, or increase in serum creatinine to ≥353.6 μmol/L, or initiation of RRT irrespective of serum creatinine | 0.3 mL/kg/h for 24 h  or anuria for 12 h |

** Rises in serum creatinine are known or presumed to have occurred in the preceding 7 days*

*RRT: Renal Replacement Therapy*

**Supplemental Table 2. Spectroscopic bioimpedance analysis**

|  | Nephrology intervention  n= 59 | Standard of care  n =54 |
| --- | --- | --- |
| Weight (Kilograms, *mean* (sd)) | 76.6 (14.5) | 75.8 (16.7) |
| NH Weight (Kilograms,*mean* (sd)) | 76.6 (14.9) | 76.5 (17.2) |
| LTI (Kilograms/m^2^,*mean* (sd)) | 14.4 (2.7) | 15.1 (3) |
| FTI (Kilograms/m^2^,*mean* (sd)) | 14.5 (5.2) | 15.1 (4.7) |
| TBW (liters, *mean* (sd)) | 35.9 (7.4) | 37.4 (8.9) |
| ECW (liters, *mean* (sd)) | 16.3 (3.2) | 17.1 (3.9) |
| ICW (liters, *mean* (sd)) | 19.5 (4.3) | 20.4 (5.2) |
| Quality (%, *mean* (sd)) | 93.4 (4.5) | 92.3 (3.4) |

*ECW: Extracellular water, FTI: fat tissue index, ICW: Intracellular water, LTI: lean tissue index, NH: normohydrated, TBW: Total body water.*

**Supplemental Table 3. Nephrology interventions definitions and accomplishment criteria.**

|  | Definition | Accomplishment criteria |
| --- | --- | --- |
| Acidosis correction | For metabolic acidosis with a pH< 7.35 and a HCO3 plasma level below 20 mmol/L, alcali supplements were precribed. | HCO3 level > 20 mmoL/L |
| Diet assessment | Evaluation of dietary intake and education was performed in case of bad habits^1^ | Improvement of dietary habits. |
| Tobacco assesment | Smoking habits were questioned, and immediate cessation of consumption was indicated. | Cessation of consumption |
| Glicaemia assessment | In the case of meeting the diabetes diagnostic criteria, treatment was iniciated. In case of previous diabetes with bad control, treatment was changed. | HbA1c ≤ 7% |
| Obesity reduction | Evaluation of dietary intake and caloric restriction and education was performed in case if BMI >30. | BMI < 30 |
| Proteinuria reduction | In case of PCR ≥ 150 or ACR ≥ 30mg/g, ACEi or ARBs were initiated. | PCR or ACR < 30mg/g |
| Salt intake assessment | Urinary sodium excretion was used to assess salt intake, if the level of sodium/creatinine ratio in spot urine was greater than 25.8 mol/mol, dietary restriction to less than 5 g per day was recommended ^2^ | Sodium/creatinine ratio in spot urine < 25.8 mol/mol |
| Diuretic adjustment | With the aim of achieving euvolaemia based on clinical data or bioimpedance parameters, the diuretic dose was titrated to the lowest effective dose or potentially discontinued. | Achievement of euvolaemia based on clinical data and/or bioimpedance parameters |
| Statin adjustement | In the case of LDL cholesterol level ≥ 100mg/dl or HDL ≤ 40mg/dL, treatment with statin was iniciated. In case of previous dyslipemia with bad control, treatment was changed ^3^ | LDL cholesterol level ≤ 100mg/dl or HDL ≥ 40mg/dL |
| ACEi or ARBs adjustment | If self-measurement of blood pressure levels were >145/85mmHg or proteinuria was detected, the treatment was initiated or increased.  If self-measurement of blood pressure levels were <110/60mmHg, the treatment reduced or withdrawn. | Treatment adherence and achievement a level of self-measurement of blood pressure levels between 145/85 and 110/60mmHg |
| Other antihypertension drugs adjustment | Based on blood pressure levels if ACEi or ARBs could not be adjusted the treatment was modified:  - in case of >145/85, treatment was increased.  - in case of <110/60, treatment was decreased | Treatment adherence and achievement a level of self-measurement of blood pressure levels between 145/85 and 110/60mmHg |
| Antiplatelet adjustment | Evaluation of compliance with antiplatelet therapy if indicated. In case of non-compliance, reinforcement of the need to take the drug. | Treatment adherence |
| Hypouricemia drugs initiation | If serum uric acid values were ≥ 7.2 mg/dL, hypouricemia drugs were initiated | Serum uric acid ≤ 7.2 mg/dL |
| NSAID withdraw | NSAID use was questioned, and immediate cessation of consumption was indicated. | Cessation of NSAID consumption |
| Anemia assessment | If:  - hemoglobin (Hb) levels were <8 g/dL, blood transfusion was prescribed,  - ferritin levels were ≤ 300 mcg/L with passing saturation of less than 20% and Hb ≥ 10g/dL oral iron therapy was initiated,  - ferritin levels were ≤ 300 mcg/L with passing saturation of less than 20% and Hb ≤ 10g/dL intravenous iron therapy was prescribed.  * Identification and correction of hematinic deficiencies (iron, B12, or folate) or hypothyroidism, if present, was the first step ^4^ | Hb≥12 g/dL and ferritin levels ≥ 300 mcg/L with passing saturation ≥ 20%, |

ACEi: angiotensin converting enzyme inhibitor; *ACR: Albumine-to-creatinine ratio;* ARBs: angiotensin II receptor blocker; BMI: Body Mass Index; HDL: High Density Lipoprotein Cholesterol; LDL: Low Density Lipoprotein Cholesterol; *NSAID: Non-steroidal anti-inflammatory drug; PCR: Protein-Creatinine-Ratio*

**Supplemental Table 4. Comparison of measured variables before and after Nephrology intervention.**

|  | Standard of care | | Nephrology Intervention | | Mean of differences from Randomization | | | |
| --- | --- | --- | --- | --- | --- | --- | --- | --- |
|  | Randomization | Hospital Admission | Randomization | Hospital Admission | Standard of care | Nephrology Intervention | p-value^1^ | Effect Size [CI95%]^2^ |
| Weight (Kg, mean (sd)) | 76,86 (13.7) | 75,8 (16.7) | 76.3 (13.9) | 76.6 (14.5) | 0.01 (6.79) | -0.60 (4.07) | 0.571 | 0.02 [-0.25;0.28] |
| BMI (Kg/m^2^, mean (sd)) | 28.0 (3.9) | 27.6 (4.4) | 28.1 (4.0) | 29.0 (4.4) | 0.08 (2.35) | -0.20 (1.42) | 0.449 | 0.01 [-0.25;0.28] |
| Serum creatinine (mmol/L, mean (sd)) | 89.0 (29.9) | 83.9 (28.1) | 89.6 (27.8) | 85.6 (28.7) | -4.70 (14.9) | -4.49 (13.5) | 0.885 | 0.16 [0.01;0.31] |
| Urine protein-Creatinine-Ratio (mg/g, median (IQR)) | 2.1 [0.9;5.3] | 2.1 [0.8;4.2] | 3.2 [1.3;7.6] | 2.1 [1.00;3.8] | -14.35 (67.5) | -1.38 (12.4) | 0.078 | 0.23 [0.02;0.44] |
| Total cholesterol (mmol/L, mean (sd)) | 4.5 (1.1) | 3.9 (1) | 4.5 (0.9) | 3.9 (0.9) | -0.57 (0.80) | -0.52 (0.82) | 0.664 | 0.55 [0.36;0.75] |
| LDL cholesterol (mmol/L, mean (sd)) | 2.4 (0.8) | 2.1 (0.9) | 2.7 (1) | 2.1 (0.8) | -0.55 (0.92) | -0.29 (0.75) | 0.042 | 0.49 [0.28;0.7] |
| Uric acid (mmol/L, mean (sd)) | 352 (99.1) | 344 (97.0) | 354 (95.6) | 343 (97.8) | -6.80 (62.5) | -2.74 (63.5) | 0.652 | 0.05 [-0.15;0.25] |
| Hemoglobin (g/L, mean (sd)) | 140 (17.1) | 130 (17.7) | 141 (15.8) | 133 (16.9) | -7.90 (13.3) | -9.30 (13.0) | 0.333 | 0.51 [0.35;0.66] |
| Ferritin (ng/mL, median (IQR)) | 118 [64.7;202] | 106 [58.2;175] | 128 [65.2;227] | 116 [67.7;227] | -12.37 (127) | -22.84 (62.3) | 0.483 | 0.12 [-0.09;0.32] |
| Hba1c (%, mean (sd)) | 5.9 (0.9) | 5.9 (0.9) | 5.9 (0.9) | 5.9 (0.8) | -0.08 (0.35) | 0.02 (0.58) | 0.157 | 0.03 [-0.19;0.24] |

** Hba1c: Glycated hemoglobin; LDL: Low Density Lipoprotein Cholesterol; SD: standard deviation*

*^1^T-Test, ^2^ Cohen’s d*

**References**

1. Waxman A. WHO global strategy on diet, physical activity and health. *Food Nutr Bull*. 2004;25(3):292-302. doi:10.1177/156482650402500310
2. Jackson SL, Cogswell ME, Zhao L, Terry A, Wang C, Wright J, et al. Association between urinary sodium and potassium excretion and blood pressure among adults in the United States: National Health and Nutrition Examination Survey , 2014. *Ciruclation* 2019;137(3):237-246. doi:10.1161/CIRCULATIONAHA.117.029193.Association
3. Catapano AL, Graham I, De Backer G, Wiklund O, Chapman JM, Drexel. H, et al. 2016 ESC/EAS Guidelines for the Management of Dyslipidaemias. *Eur Heart J*. 2016;37(39):2999-3058l. doi:10.1093/eurheartj/ehw272
4. Anand IS, Gupta P. Anemia and Iron Deficiency in Heart Failure: Current Concepts and Emerging Therapies. *Circulation*. 2018;138(1):80-98. doi:10.1161/CIRCULATIONAHA.118.030099
